# Drawing techniques as tools for the evaluation of scholastic integration and emotional components in primary and secondary school: A cross-sectional study

**DOI:** 10.3389/fpsyg.2022.1046626

**Published:** 2023-01-19

**Authors:** Sabina La Grutta, Marco Andrea Piombo, Martina Riolo, Vittoria Spicuzza, Umberto Maria Cianciolo, Federica Andrei, Elena Trombini, Maria Stella Epifanio

**Affiliations:** ^1^Department of Psychology, Educational Science and Human Movement, University of Palermo, Palermo, Italy; ^2^Department of Psychology “Renzo Canestrari”, Alma Mater Studiorum – University of Bologna, Bologna, Italy

**Keywords:** scholastic integration, projective graphic techniques, drawn stories technique, classroom draw, emotional-affective components, primary school, secondary school

## Abstract

**Introduction:**

In the last decades, many studies have emphasized emotion’s role in psycho-educational processes during childhood, such as scholastic integration. Emotional variables in childhood can be assessed through projective graphic techniques, as they allow children to use kinetic components of the draws to communicate emotions.

**Method:**

1.757 couple of draws were collected, from primary school children (*N* = 1.270; *F* = 643 [50.6%]; Age = 8.6; SD = 1.31) and secondary school children (*N* = 487; *F* = 220 [45.2%]; Age = 11.72; SD = 0.70) and from eight schools in Sicily and over 60 different classrooms. The Drawn Stories Technique and the Classroom Draw were used to assess children’s current emotional state and scholastic integration.

**Results:**

Pearson’s correlation showed significant relationships between the Drawn Stories Technique and both sex and age. In contrast, Classroom Drawing total score showed a significant relationship with the female sex but no significant relationship with age. Linear regression analysis, including sex and age as independent variables, showed that sex is a significant predictor of Negative Outcomes of the Drawn Stories Technique, while no effect of age was detected.

**Discussion:**

These findings showed that adequate attention is needed to the learners’ emotional-affective world that influences their relationships and their vision within the class group. Although the drawing techniques alone seem to be not as such sufficient to explain children’s individual differences in the classroom on the whole, they could be helpful for the teacher to facilitate dialogues with children, modulate didactical materials, and detect and prevent some problems in group class functioning.

## 1. Introduction

Since the first development of psychology, drawing has been considered a useful tool to understand an individual’s development and personality (Driessnack, 2005). Projective techniques based on drawing acquired ever more popularity among clinicians because of their simple administration and ease of acceptance, especially by children (Gross and Hayne, 1998). The graphic method is considered a useful way to express not only personality dimensions but also the child’s emotions, and the affective tone with which children “emotionally invest” the context in which they live (Longobardi et al., 2017).

In the last decades, many studies have shown the fundamental role of emotions on psycho-educational processes during childhood and how good emotion management can be a pathway to better social competence in future (La Grutta et al., 2022). Particularly, current evidence shows that children may be able to express emotions through drawings even if they are unable to communicate or express them verbally (Fury et al., 1997; Malchiodi, 1998; Kim and Suh, 2013; Pace et al., 2013; Goldner et al., 2015). Some others have also suggested that, through their drawings, children can create connections that reveal their own mental internal world (Cox, 2013). For these reasons, drawing could be the best way for children to communicate their feelings, conflicts, and mental states, and it is halfway between acting and dreaming (Cox, 2013). Therefore, graphic techniques are an important assessment tool, capable of providing new knowledge about children’s intellectual development, emotional dimensions, and personality traits.

Moreover, based on the dynamic and esthetic qualities perceived in drawings, we can identify various developmental stages in the drawing: 4-year-old children start to draw a more accurate human figure (e.g., gender differences are included), from 6 to 7 years, there are even more details (e.g., the ground line and decorative intent) and also appear text in balloons, and at 8–9 years, children start to use transparency, aerial point of view, perspective, and movement until pre-adolescence in which draws are similar to that in adulthood in accuracy (Quaglia et al., 2013; Scafidi Fonti et al., 2015).

Drawing could also reflect, through some emotional indicators, gender differences in emotional expression, and conflictual themes, which could be different due to biological and cultural factors. For example, males could tend to use more externalization strategies to express their anxiety or conflictual themes in more aggressive manners (broken lines, more deletions, and paper ripped off) than females who could use more internalization strategies (e.g., depressive contents in the draws, blame if they are not able to draw properly; Picard and Boulhais, 2011; Chaplin, 2014; Scafidi Fonti et al., 2015).

According to that, in a clinical context, two of the most frequently used drawing techniques are the Draw-a-Person Test and the Family Drawing Test (Goodenough, 1926; Machover, 1953; Hammer, 1958; Harris, 1963; Corman, 1967), both widely employed in a psychodiagnostic assessment (Skybo et al., 2007). Particularly, the Draw-a-Person Test enables the clinician to capture the child’s perception of their own self and to release their private fantasies and anxieties (Machover, 1949), and the perception helps to understand children’s representations of their parents (McGuigan and Pratt, 2001; Piperno et al., 2007).

In clinical practice with children, besides these two techniques, spontaneous drawing has always been widely used (Trombini et al., 2004). An example of a graphic technique that is based on both free drawings and narration is the “Drawn Stories Technique” (Trombini, 1994), which was developed originally in a psychoanalytic and psychodiagnostic context to facilitate not only empathic communication and narrations with patients but also the evaluation, detection, and interpretation of psychological suffering in developmental age. This technique permits the expression of free drawing in a sequence of scenes and encourages the construction of many possible narrative developments. The conclusions from these can be evaluated according to well-defined categories, such as the outcome of the story, which expresses the levels of emotional distress of children (Trombini et al., 2001). The psychologist asks a child to draw an invented story, without insisting on any point of view and waiting for the child to draw the story. Through this technique, children can express their affective themes and internal conflicts. These stories can be classified depending on how the story ends: (1) Positive Outcome (PO): the subject ends his narration positively without any accident. (2) Negative Outcome (NO): the subject ends their narration negatively with an accident; (3) Compensated Positive Outcome (CPO): it signed when the story, despite the presence of an accident, ends positively; (4) Absent Outcome (AO): the story is not completed. In particular, in a study conducted by Trombini et al. (2004) on an Italian sample of 211 primary and secondary school children, this technique showed good validity in detecting anxiety and depression through negative outcomes in the stories.

**Figure 1:**
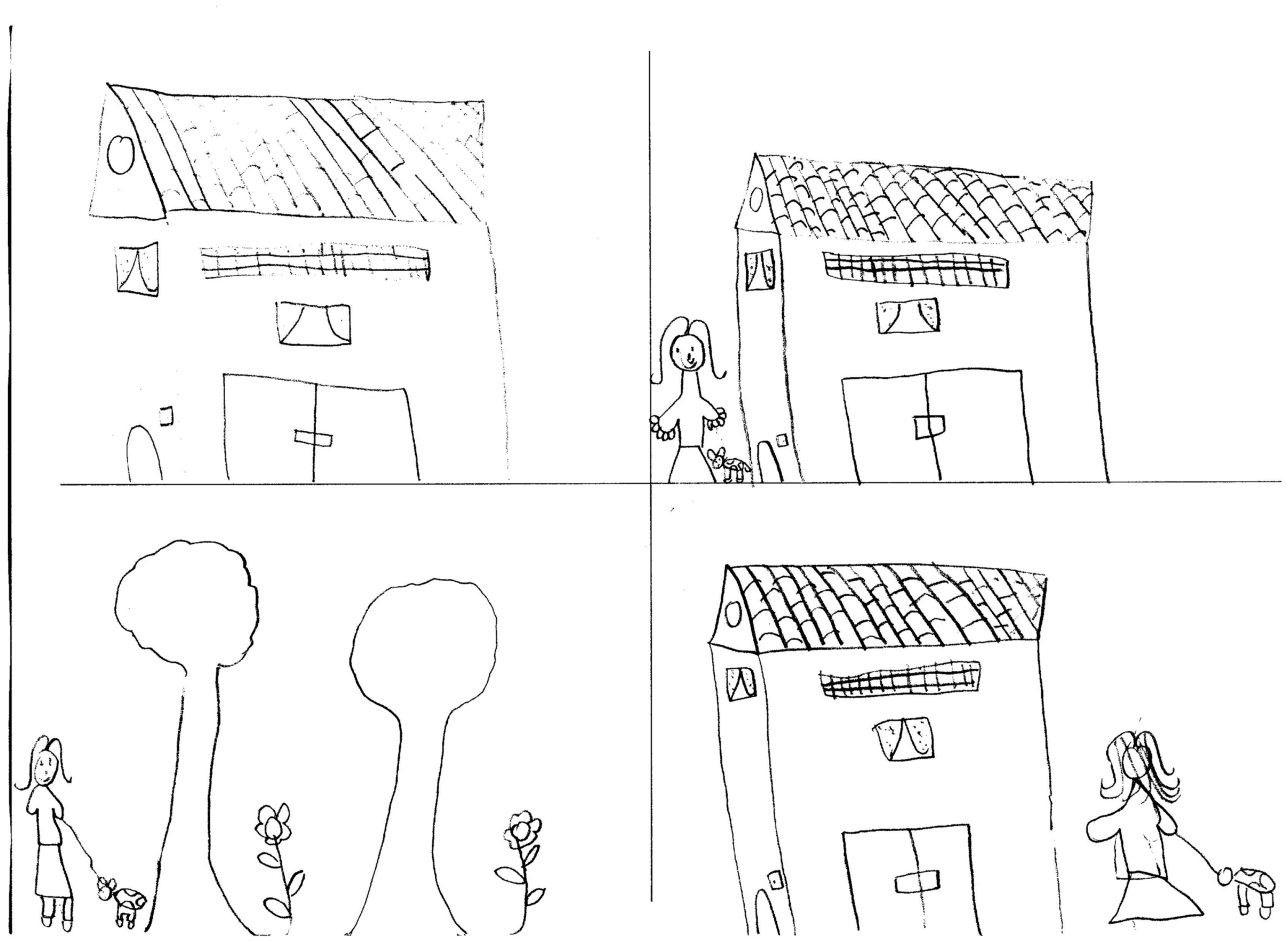
Drawn Stories Technique. Positive outcome (PO) example: “*There is a girl in the house. Then, she goes out to walk the dog. Finally, she returns to the house and she is happy about that beautiful walk.*” Female, 8  years old.

**Figure 2:**
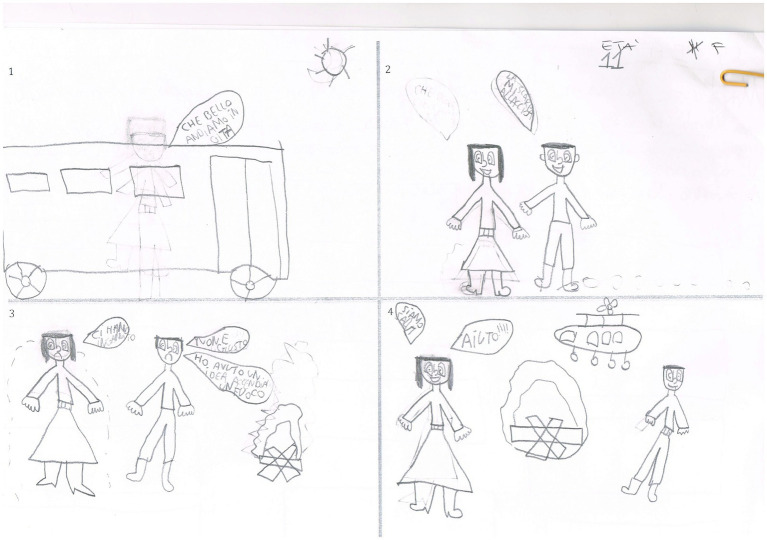
Drawn Stories Technique. Positive compensated outcome (PCO) example: “*During a school trip, two scholars stop to look at the landscape but are abandoned by the rest of the class. Finally, they light a fire and have been saved with an helicopter.”* Male, 11  years old.

**Figure 3:**
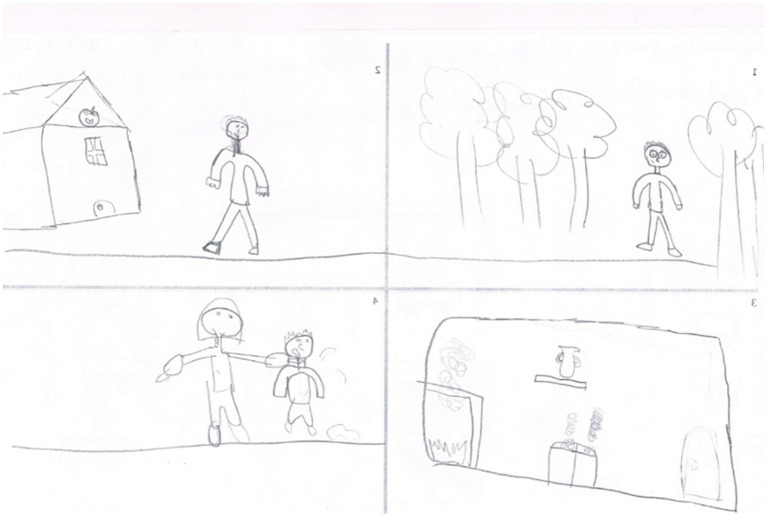
Negative outcome (NO) example: “*There is a child that is walking in the woods. He sees an house, due to his curiosity enters in that but he’s been killed by a man who’s hiding there.*” Male, 10 years old.

**Figure 4:**
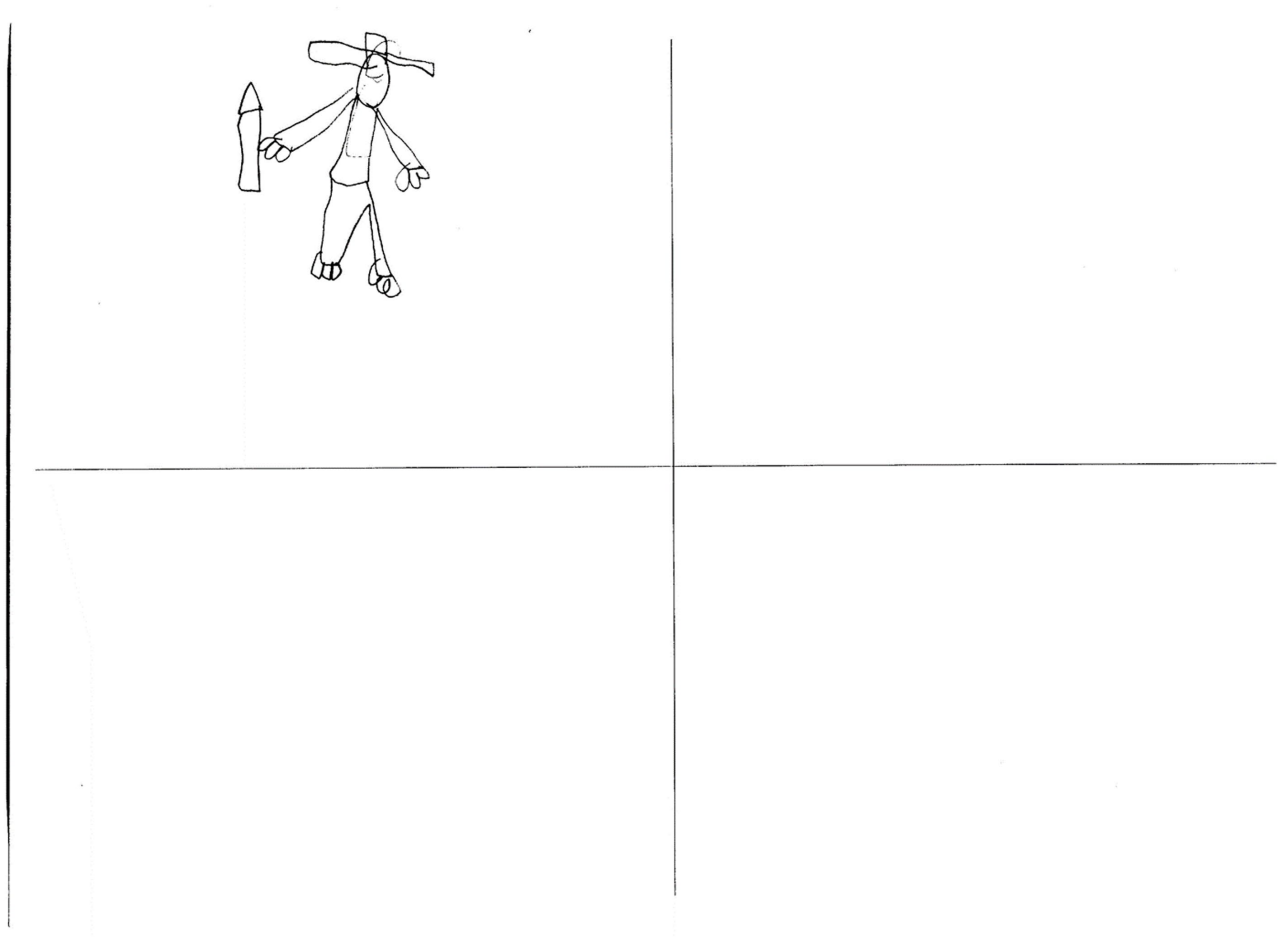
Absent outcome (AO) example: “*There is a man…. and then… I do not know.*” Female, 9 years old.

Moreover, clinical practice and a number of studies show that these types of endings indicate the emotional state of the drawer. In particular, PO and CPO indicate an emotional wellbeing and resilience capacity (Figures 1, 2), NO indicates an emotional turbulence that could be related to aggressive, anxious, or depressive themes (Figure 3), while AO can indicate a block of symbolic expression (Scafidi Fonti et al., 2015; Figure 4).

This could be possible because drawing is most beneficial for school-age children because their cognitive thinking is primarily concrete but develops an understanding of abstract concepts. As cognition develops, adolescents become more resistant to drawing and find it easier to express their feelings in words, music, or physical activity (Skybo et al., 2007). Furthermore, drawings are often called upon by professionals as a method of allowing a child to communicate more freely, with no language being necessarily involved, as well as a way of “breaking the ice” between the child and professional (Veltman and Browne, 2002).

Schools are often the primary context where children have acceptance or refusal experiences with their peers (Rubin et al., 2007). In such a context, the emotional development of children could promote their ability to manage the needs of their social and educational environments, keep good relationships with peers, recover from negative emotions, tolerate frustrations, express emotions in adaptive manners, and improve the processes of integration.

In light of this, in such psycho-educational contexts, the use of drawing tools, such as the Drawn Stories Technique integrated with other instruments that are more specific for the scholastic context such as “The Classroom Drawing” (Quaglia and Saglione, 1990), could be an important way teachers have to drive classroom relationships, to facilitate a good affective atmosphere, providing to the pupils a way to learn expressing their emotional states, positive or negative ones, to understand owns and others, to assess the quality of relationships among child, teachers, and classmates, and to evaluate scholastic integration level (Scafidi Fonti et al., 2015).

The classroom drawing is designed to investigate the child’s perception of their “feeling good” at school, in terms of classroom integration such as the relationship with the teachers and classmates and the experience of learning and of him/herself as a pupil (Quaglia and Saglione, 1990).

Starting with growing interest in emotional education in Italian schools, the emotional factors that are fundamental in social interaction have been studied with increasing interest, especially regarding their effect on scholastic integration.

In particular, social–emotional competence could be considered a critical factor to target with universal preventive interventions that are conducted in schools because the construct associates with social, behavioral, and academic outcomes that are important for healthy development; predicts important life outcomes in adulthood; can be improved with feasible and cost-effective interventions; and plays a critical role in the behavior change process (Domitrovich et al., 2017; La Grutta et al., 2022).

The aim of this study is to show how the “Drawn Stories Technique” and the “Classroom Drawing” can be considered useful tools to assess children’s emotional state within the class group and their scholastic integration in an educational context.

Specifically, the main hypotheses of the present study are as follows:

1. There are significant gender differences in the way children express their conflict and emotion through the draws, particularly males would tend to express more aggressiveness than females. Thus, a higher number of Negative Outcomes are expected for males compared with females.
2. There is a positive correlation between the Drawn Stories Technique scores and children’s age. Particularly, older children will tend to draw more Compensated Positive Outcomes due to the progressive complexity of emotional experiences and growing resiliency during their development.
3. The quality of scholastic integration assessed by the classroom drawing is positively related to age in primary school and negatively in secondary school, especially as regards the relationship with the teacher (authority).

## 2. Materials and methods

### 2.1. Procedures

The selection of schools was based on previous work relationships with schools to collect a convenience sample. Participants were recruited from eight schools in Sicily and over 60 classrooms from 2014 to 2020. Two researchers per class administered the two projective drawing techniques mentioned earlier during the school timetable and in the usual classroom. The completion time lasted approximately 45 min. The drawings were presented one by one to children as activities, without any vote or ratings, and they were motivated by the researchers: “*it’s not important how you draw, but we are interested in the stories that you want to share with us.*” Once the children finish their drawings, in turn, the researchers conducted individual brief interviews asking some simple questions such as the following: “*who are the main character of this story”? “What is its name”? “If you have to choose a character, in this story, that looks more like you, what character you choose”? “How the story ends”?* At the beginning of the school year, school principals, teachers, and parents signed the informed consent sheets about the purposes of the research and data collection procedures. Written consent was signed and collected by both parents of every child involved in the study. The study was run in accordance with the national ethics guidelines and in line with the Declaration of Helsinki. This study was approved by the University of Palermo Ethics Committee (no. 83/2022).

### 2.2. Participants

The research involved a total of 1,757 children with an age range from 6 years to 13 years from primary (*N* = 1,270; *F* = 643 [50.6%]; age = 8.26; SD = 1.31; age range 6–10 years) and secondary school (*N* = 487; *n* female = 220 [45.2%]; age *M* = 11.72; SD = 0.70; age range 11–13 years) in Sicily. Neither of the participants had special educational needs (SENs) while three participants were deaf and five had autism spectrum disorder. However, all of them were able to finish their drawings.

### 2.3. Measures

#### 2.3.1. Demographics

Demographic data were taken from class registers according to parents’ permission obtained by informed consent. These data were treated and coded to ensure anonymity.

#### 2.3.2. Emotional state

To evaluate the emotional state of children, the previously described “Drawn Stories Technique” was used. The psychologist asks a child to draw an invented story, without insisting on any point of view and waiting for the child to draw the story. After the drawing phase, children are asked to write the story behind the sheet, and then, they are briefly interviewed by the researcher about their stories. In this way, it is possible to determine which character the child identifies with and to score the type of outcome based on what happens to the chosen character.

#### 2.3.3. Scholastic integration

**Figure 5:**
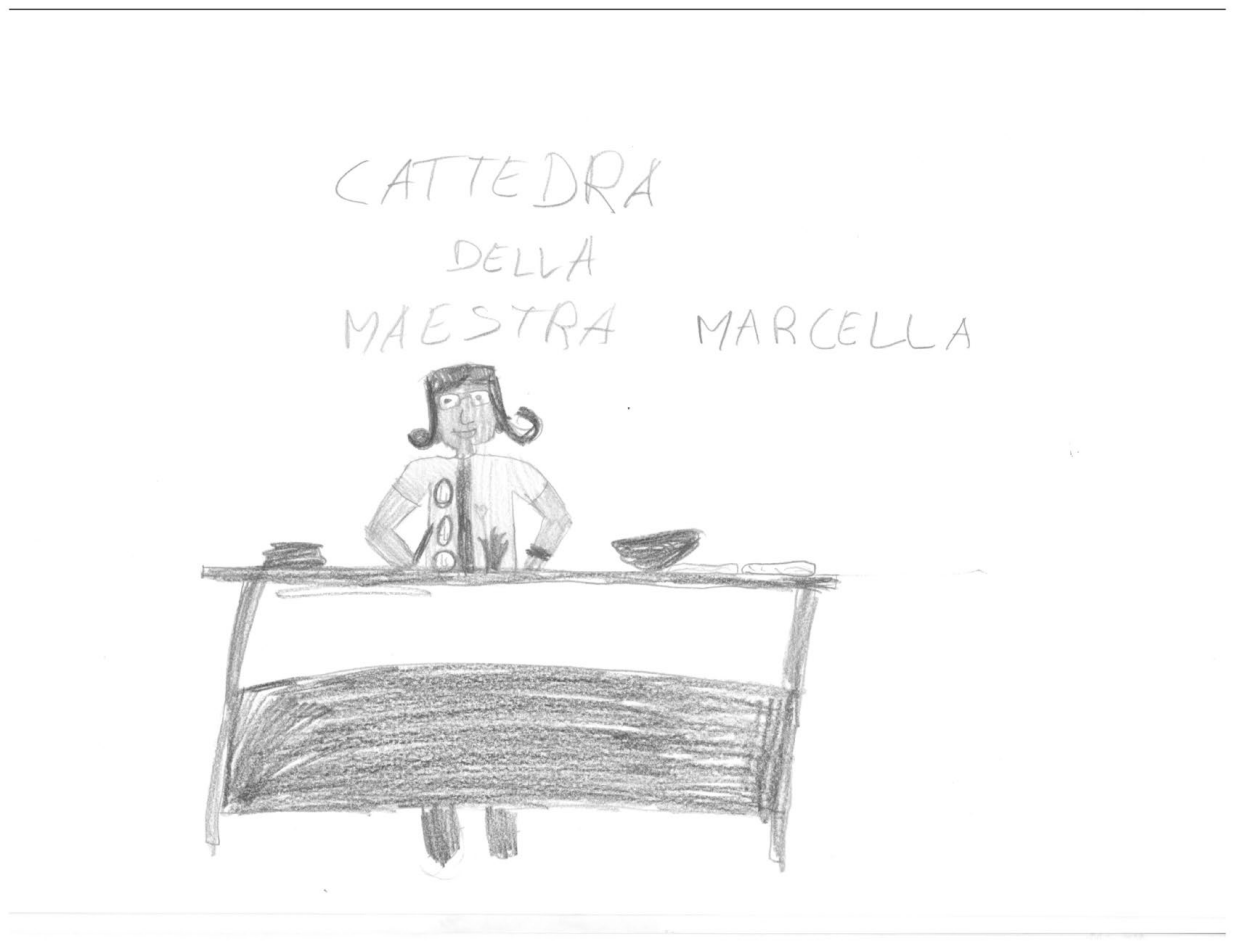
Classroom drawing: the presence of the teacher: “*The Teacher Marcella and her desk.*” Female, 10  years old.

**Figure 6:**
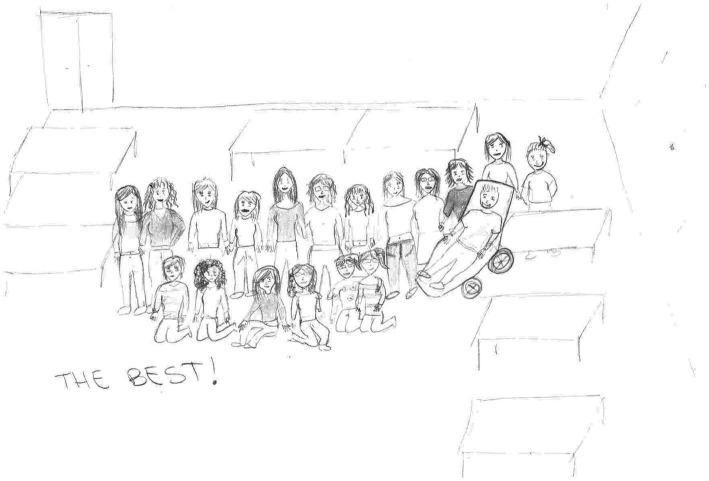
Classroom drawing: Group class and teachers. The drawer, the classmates, and the teacher. Integration 3. Female, 12  years old.

**Figure 7:**
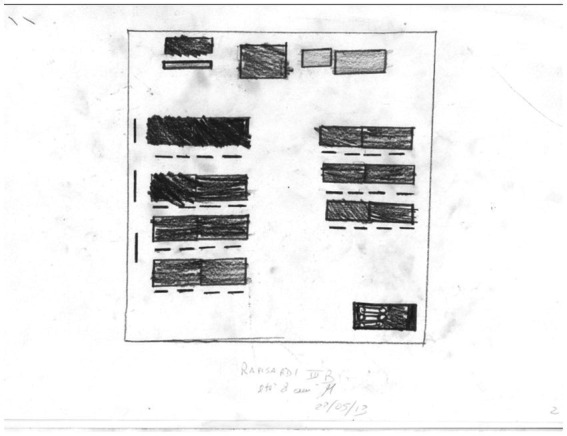
Classroom drawing: an empty class example, integration 0. Male, 8  years old.

To evaluate scholastic integration, “The Classroom Drawing” was used. Children are asked to draw their class in whatever way they like. The analysis of the drawing takes into account the presence or absence in the drawing of (1) the teacher (relationship with authority, Figures 5, 6); (2) classmates (level of socialization); and (3) the drawer themselves (personal involvement in the class). Each of these elements is scored as dichotomous variables: 0 means their absence (Figure 7), while 1 indicates their presence in the drawing. Their sum provides a global classroom integration index, which therefore ranges from 0 (Figure 7) to 3 (Figure 6), with 3 indicating more adaptive integration levels (Quaglia and Saglione, 1990).

### 2.4. Data analysis

Statistical analyses were performed using programs available in the Statistical Package for Social Sciences (SPSS for Windows release 25.0). Descriptive statistics were utilized to describe the data (frequencies, percentages, mean, and standard deviation). Moreover, two analyses of variance (ANOVA) were performed in which the outcomes of the Drawn Stories Technique and Classroom Drawing scores were used as the dependent variables. In both cases, gender (male vs. female) and school level (primary vs. secondary) were included as dichotomous factors. Gender and the outcomes of the Drawn Stories Technique were coded as a dummy variable: *F* = 0 and M = 1 for gender and AO = −1, NO = 0, PO = 1, and CPO = 2 for the Drawn Stories Technique, respectively. Finally, multiple linear regression analyses were performed to test the predictive capacity of gender and age for two specific scores such as Negative Outcomes in the Drawn Stories Technique and the presence of teacher in the Classroom Drawing treated as dichotomous variables 0–1(absence–presence).

## 3. Results

### 3.1. Drawn Stories Technique outcomes by level of education and gender

Regarding the Drawn Stories Technique, Table 1 shows descriptive statistics of the whole sample with a prevalence of CPO (44.3%), followed by PO (39.2%), NO (14.1%), and AO (2.3%). Using Drawn Stories Technique outcomes as a function of the level of the school, we found that there is a prevalence of PO (45.7%) in primary school children, followed by CPO (39.7%) and NO (11.7%), and AO was reported only in 2.9% of cases. In comparison, in secondary school, the most recurring outcome is CPO (56.5%), followed by PO (22.4%) and NO (20.3%), and AO was reported only in 0.8% of cases.

**Table 1:** Frequencies of Drawn Stories Technique outcomes and classroom drawing scores.

		Primary		Secondary		Total
		Female	Male	Female	Male	Sample
DST	AO	1.2%	4.6%	1.8%	0.0%	2.3%
	NO	6.4%	17.2%	14.1%	25.5%	14.1%
	PO	45.7%	45.6%	24.1%	21.0%	39.2%
	CPO	46.7%	32.5%	60.0%	53.6%	44.3%
CD	0	39.3%	44.3%	48.2%	53.6%	44.4%
	1	12.6%	16.3%	9.1%	5.2%	12.4%
	2	22.4%	16.7%	19.5%	19.1%	19.5%
	3	25.7%	22.6%	23.2%	22.1%	23.7%
TEACH	1	59.6%	65.1%	66.4%	71.8%	62.3%
	0	40.4%	34.9%	33.6%	28.2%	37.7%

DST, Drawn Stories Technique; CD, Classroom drawing; TEACH, Presence of the teacher in classroom drawing; NO, Negative outcome.

*p < 0.05, **p < 0.01, and ***p < 0.001.

Moreover, the results, including also gender comparisons, showed that females, both in primary school and secondary school, reported higher CPO (46.7% and 60%, respectively) than males in primary and secondary school (32.5% and 53.6%, respectively); PO is essentially balanced between females and males both in primary (45.7% and 45.6% respectively) and secondary school(24.1% and 21% respectively). In contrast, males show higher NO than females both in primary(17.2% and 6.4%) and secondary school(25.5% and 14.1%) (Table 1).

Moreover, to test the hypothesis, ANOVA was performed, and NO was changed into dichotomous and discrete values (0–1). The results showed significant differences between primary and secondary school children in NO (*F* = 21.69 *p* < 0.01) with a small effect size (*d* = 0.21), and also gender differences in NO were significant both in primary (*F* = 37.073; *p* < 0.01;) with a small effect size (*d* = 0.34) and secondary school children (*F* = 9.794; *p* < 0.01) also with small effect size (*d =* 0.27; Table 2).

**Table 2:** Descriptive statistics of quantitative drawing indices and differences by sex and type of school.

	Primary		Secondary	
	Female	Male	Female	Male
DST	1.38 ± 0.66	1.06 ± 0.825	1.42 ± 0.79	1.28 ± 0.84
ANOVA	*F* = 8.829**	0.003	*F* = 3.570	0.059
NO	0.06	0.17	0.14 ± 0.34	0.25 ± 0.43
ANOVA	*F* = 21.698***	0.000	*F* = 9.794**	0.002
CD	1.34 ± 1.23	1.18 ± 1.22	1.18 ± 1.25	1.10 ± 1.26
ANOVA	*F* = 3.752	0.053	*F* = 0.483	0.488
TEACH	0.40 ± 0.49	0.35 ± 0.47	0.34 ± 0.47	0.28 ± 0.45
ANOVA	*F* = 7.647**	0.006	*F* = 1.676	0.196

DST, Drawn Stories Technique; CD, Classroom drawing; TEACH, Presence of the teacher in classroom drawing; NO, Negative outcome.

### 3.2. The classroom drawing scores by level of education and gender

Regarding the content of the Classroom Drawing, descriptive statistics of the total sample showed a prevalence of low scores of integration, such as 0 (44.4%) and 1 (12.4%), followed by good scores of integration 2 (19.5%) and best scores with 3 (23.7%).

Using Classroom Drawing scores as a function of the level of education, the results show that in primary school children, low scores such as 0 and 1 are reported in 41.8 and 14.4% of cases, respectively. In comparison, higher scores such as 2 and 3 are reported in 19.6 and 24.2% of cases, respectively. Moreover, in secondary school, there is a prevalence of low scores, such as 0 (51.1%) and 1(7%), while higher scores such as 2 and 3 are reported in 19.3% and 22.6%, respectively.

To test, HP3 ANOVA was performed first with total Classroom Drawing mean scores and second taking into account only the relationship with the teacher as a separate dichotomous variable (0–1). The results reported in Table 2 show that primary school children score better than secondary school children. However, this difference is only nearly significant (*F* = 3.752; *p* > 0.05) with a small effect size (*d =* 0.10), whereas, as regards the relationship with teacher score, significant differences were found with primary school children that score better than secondary school children (*F* = 7.647; *p* < 0.01) with small effect size (*d* = 0.14). Regarding gender comparison, total female scores are significantly better than males in primary school (*F* = 5.847; *p* < 0.05) but not significantly better in secondary school children (*F* = 0.483; *p* > 0.05). Finally, also regarding teacher relationship score, females score significantly better than males in primary school (*F* = 4.105; *p* < 0.05) but not in secondary school (*F* = 1.676; *p* > 0.05).

### 3.3. Correlation analyses

Regarding bivariate associations in the total sample, Pearson’s correlation showed significant relationships between the Drawn Stories Technique and both sex (*r =* 0.168; *p* < 0.01) and age (*r* = 0.69 *p* > 0.01), while it showed no significant relationship with Classroom Drawing scores (*r* = −0.016; *p* > 0.05; Table 3). Regarding Classroom Drawing total score, Pearson’s correlations show a significant relationship with the female sex (*r* = −0.060 *p* > 0.05) but no significant relationship with age (*r* = −0.14; *p* > 0.05; Table 3).

**Table 3:** Correlation analysis.

	2	3	4	5	6
1. SEX	0.16	−0.168**	,060*	-,060*	0.163***
2. AGE		0.69**	−0.014	−0.13	0.081**
3. DST			0.016	−0.017	−0.650**
4. CD				0.777*	0.010
5. TEACH					0.004
6. NO					

*p < 0.05, **p < 0.01, and ***p < 0.001.

### 3.4. Regression analyses

In the sample of primary school, linear regression analysis performed selecting specifically “Negative Outcome” (NO) as the dependent variable showed that the model, which includes sex and age as predictors, explained a total of 2.8% (*F* = 19.037; *p* < 0.001) of variance with sex as only significant predictor of negative outcomes (*β* = 0.167; *p* < 0.001) in primary school (Table 4).

**Table 4:** Linear regression model: sex and age as predictors of negative outcomes in primary and secondary school children.

Primary	*B*	St.Err.	Beta	*R* ^2^	Adj.*R*^2^	*t*	Sign.
Model	0.120	0.058		0.029	0.028	2.080	0.038
SEX	0.108	0.018	0.167			6.045***	0.000
AGE	−0.007	0.007	−0.028			−1.000	0.318
Secondary							
Model	−0.372	0.305				−1.221	0.223
SEX	0.116	0.036	0.144	0.026	0.022	3.197***	0.001
AGE	0.044	0.026	0.076			1.689	0.092

Regarding the secondary school, the model explained only 2.2% of variance with sex as the only significant predictor (*β =* 0.116; *p* < 0.001).

Regarding the presence of the teacher in the Classroom Drawing as a dependent variable, regression analyses for the sample of the primary school revealed only 0.5% of the variance (*F* = 3.083; *p* > 0.05) with sex as the only significant predictor *(β* = −0.165 *p* < 0.000). In the sample of secondary school, the model revealed only 1.5% of the variance (*F* = 4.698; *p* < 0.01) with age as the only significant predictor (*p* < 0.001; Table 5).

**Table 5:** Linear regression model: sex and age as predictors of teacher presence in Classroom Drawing scores in primary and secondary school children.

Primary	*B*	St. Err.	Beta	*R* ^2^	Adj. *R*^2^	*t*	Sign.
Model	1.220	0.224		0.005	0.003	5.454	0.000
SEX	−0.165	0.069	−0.067			−2.393*	0.017
AGE	0.015	0.026	0.016			0.569	0.570
Secondary							
Model	−1.672	0.959		0.019	0.015	−1.745	0.082
SEX	−0.067	0.114	−0.026			−0.587	0.558
AGE	0.243	0.081	0.134			2.984**	0.003

## 4. Discussion

The current study aimed to explore how graphic techniques evaluate children’s emotional state and scholastic integration and contribute to a growing literature on the role of emotion-related attributes on psycho-educational processes during childhood, such as scholastic integration. According to our first hypothesis, there is a significant gender difference in the way children express their emotions; specifically, our findings show that males tend to draw a greater number of NO than females in the Drawn Stories Technique, and this tendency is stronger for primary than secondary school children, as confirmed by the only previous research, which used this technique so far conducted by Trombini et al. (2004). According to our findings, a meta-analysis by Chaplin and Aldao (2013) found that gender differences in many of the emotion expressions either diminished (for internalizing emotion expressions) or reversed direction (for externalizing and negative emotion expressions) in adolescence; for authors, it is possible that physiological (e.g., puberty) and social (e.g., at school and in the peer group) changes in adolescence lead to an increase in internalizing emotional expressions for both boys and girls, attenuating gender differences for this emotion category (Chaplin and Aldao, 2013). Overall, this gender difference can be explained in different ways, taking into account biological (Zahn-Waxler et al., 2008; Connolly et al., 2019) and psychosocial factors (Wright et al., 2018; Mancini et al., 2020), but it is important to highlight that negative outcomes indicate a presence of emotional turbulence in the “here and now,” and drawing is a fundamental and also the easiest way that children have to contain and regulate emotion (La Grutta et al., 2022). Moreover, the results showed that females reported higher CPO both in primary and secondary school; increases in positive outcomes compensated and negative outcomes could be considered an evolutionary advancement reflecting children’s development as a consequence of a more complex reality and a growing resilience capacity.

Gender comparison also showed that females’ total scores are better than males in the Classroom Drawing, but this difference is significant only for primary school; this tendency also regards teacher relationship score in which females score significantly better than males in primary school but not in secondary school. These findings support previous research (Baker, 2006; Quaglia et al., 2013; Longobardi et al., 2016) in which the association between teacher relationship quality and the pupil’s sex seems to be higher for females and could reflect the teachers’ tendency to find less cohesion and affinity in relationships with male pupils that could be connected to the boys’ lack of faith in their mental abilities and their difficulty in responding easily to the cognitive demands made by the teacher in terms of effort and scholastic achievement (Longobardi et al., 2016). Another possible explanation is related to the fact that primary school teachers in Italy are primarily females, and this could have an important role in facilitating some identification by girls with them.

The Classroom Drawing seems to reflect children’s individual differences only marginally, maybe due to the complexity of the school environment in which some variables such as teachers’ educational styles and the physical spaces of schools could influence the current evaluation (Brunetti et al., 2020). Despite this, the classroom drawing is, therefore, an important evaluation tool to assess the teacher–pupil relationship that is regarded as one of the fundamental modes of expression of a bond of crucial importance for the child’s emotional and cognitive development (Quaglia et al., 2013; Mancini et al., 2020). The degree of negativity in the relationship with the teacher is associated with poor academic and social behavior and prospectively through secondary school (Baker, 2006). Regarding the age variable, our findings show a negative correlation between secondary school and level of integration, especially for the relationship with the teacher; these results suggest that the development and differentiation of cognitive and self-system processes may decrease the prominence of teacher–child relationship by secondary school when children report less positive relationships with teachers and more investment in peer relationships (Baker, 2006). Moreover, the relationship with the teacher represents the relationship with authority and it is possible that the transition period from childhood to adolescence, which involves an increase in conflicts toward authority and social norms in favor of achieving greater autonomy, could lead to a worse relationship with the teacher as a representation of authority (Smetana et al., 2005).

## 5. Limitations

Our study suffers from some limitations. First, the cross-sectional research design does not allow us to analyze changes over time. Moreover, we used only projective graphic techniques, and adding different types and more objective tools such as self-report instruments could improve the validity of these findings. Despite all the above, the correspondence with the data found in the literature enabled us to confirm the usefulness of the graphic method as an instrument for the assessment and a means of gaining knowledge of the children’s emotional state and scholastic integration to improve pupil emotional health and wellbeing in terms of the social, behavioral, and academic outcomes.

## 6. Conclusion

In conclusion, our hypotheses are partially confirmed. First, as we hypothesize, gender is significantly related to the different emotional states expressed by children and predicts NO in the Drawn Stories Technique, especially the male sex. However, age showed no relationship with NO while it was related to CPO. This is probably because of the development of children who became more capable of creating more complex stories than younger ones and use resilience strategies to address their emotional problems. Our hypotheses are partially confirmed regarding scholastic integration because age was a significant predictor of better scholastic integration in secondary school but not in primary school, as we hypothesize. However, on the whole, the effect was relatively low so seems that drawing techniques alone are not sufficient to suggest and detect children’s individual differences in the classrooms. Despite this, measures of this type are economic, easy to administer, and provide a lot of information even if they are used in a group. It could be helpful to propose some simple “activities” to the children aiming to start teachers thinking about the emotional climate of their classroom to facilitate teacher and children dialogs, talking about what is happening in the class, especially if some critical events happened, such as children’s transference or bullying behaviors, but also only modulate didactical materials and detect some dysfunctional signals to prevent problems in group class functioning and promote better integration improving children scholastic wellbeing.

## Data availability statement

The raw data supporting the conclusions of this article will be made available by the authors, without undue reservation.

## Ethics statement

The studies involving human participants were reviewed and approved by Bioethic Committee University of Palermo (no.83/2022). Written informed consent to participate in this study was provided by the participants’ legal guardian/next of kin.

## Author contributions

SG, ME, ET, and FA conceptualized the study and supervised it. MP and UC were responsible for the data collection. MP, MR, and VS coded drawings. MP and UC analyzed the data. MP, MR, and VS wrote the draft manuscript. SG, ME, ET, FA, and MP revised the final manuscript. All funders should be credited and all grant numbers should be correctly included in this section.
